# Teleoperation and Visualization Interfaces for Remote Intervention in Space

**DOI:** 10.3389/frobt.2021.747917

**Published:** 2021-12-01

**Authors:** Peter Kazanzides, Balazs P. Vagvolgyi, Will Pryor, Anton Deguet, Simon Leonard, Louis L. Whitcomb

**Affiliations:** ^1^ Department of Computer Science, Johns Hopkins University, Baltimore, MD, United States; ^2^ Laboratory for Computational Sensing and Robotics, Johns Hopkins University, Baltimore, MD, United States; ^3^ Department of Mechanical Engineering, Johns Hopkins University, Baltimore, MD, United States

**Keywords:** space robotics, teleoperation, scene modeling, model-mediated control, satellite servicing

## Abstract

Approaches to robotic manufacturing, assembly, and servicing of in-space assets range from autonomous operation to direct teleoperation, with many forms of semi-autonomous teleoperation in between. Because most approaches require one or more human operators at some level, it is important to explore the control and visualization interfaces available to those operators, taking into account the challenges due to significant telemetry time delay. We consider one motivating application of remote teleoperation, which is ground-based control of a robot on-orbit for satellite servicing. This paper presents a model-based architecture that: 1) improves visualization and situation awareness, 2) enables more effective human/robot interaction and control, and 3) detects task failures based on anomalous sensor feedback. We illustrate elements of the architecture by drawing on 10 years of our research in this area. The paper further reports the results of several multi-user experiments to evaluate the model-based architecture, on ground-based test platforms, for satellite servicing tasks subject to round-trip communication latencies of several seconds. The most significant performance gains were obtained by enhancing the operators’ situation awareness *via* improved visualization and by enabling them to precisely specify intended motion. In contrast, changes to the control interface, including model-mediated control or an immersive 3D environment, often reduced the reported task load but did not significantly improve task performance. Considering the challenges of fully autonomous intervention, we expect that some form of teleoperation will continue to be necessary for robotic *in-situ* servicing, assembly, and manufacturing tasks for the foreseeable future. We propose that effective teleoperation can be enabled by modeling the remote environment, providing operators with a fused view of the real environment and virtual model, and incorporating interfaces and control strategies that enable interactive planning, precise operation, and prompt detection of errors.

## 1 Introduction

Robots can enable exploration of space beyond human limits, as they can bring nearly human-like (and sometimes super-human) capabilities in sensing and manipulation to extreme environments, and at a lower cost and reduced risk compared to missions with human crews. In addition, the robot’s operational lifetime can be designed to suit the mission. We consider applications in servicing, assembly, and maintenance to extend human capabilities into space through remote semi-autonomous teleoperation. Currently, this includes robots operating in Earth orbit, in cislunar space, or on the lunar surface, but in the future could include robots on the surface of another planet, with humans in spacecraft orbiting that planet. Essentially, we consider scenarios where the communication latency between the humans and robots is on the order of seconds or tens of seconds, rather than minutes or tens of minutes. In these cases, telepresence and teleoperation are feasible, though challenging, and provide motivation for many research efforts.

For the last 10 years, we have been conducting research to develop, evaluate, and demonstrate new technologies for telerobotic servicing of satellites on-orbit. Much of our effort has focused on one crucial step in on-orbit refueling, which is to gain access to the satellite’s fuel ports by removing a portion of the multilayer insulation (MLI, see List of Abbreviations) that protects the outside of the satellite body. The configuration of the MLI that covers the fuel ports can be significantly different between satellites, as described below, thereby requiring different cutting approaches. In addition, we considered path planning of the robot, within its confined workspace, to exchange tools and perform the tasks required for satellite refueling.

This paper describes a model-based system architecture that enables semi-autonomous teleoperation, where the models can improve the visualization and situation awareness of the operator, provide assistance during teleoperation, and interpret sensor feedback to detect potential task failures or update the models. We report the results of experiments performed to evaluate elements of this architecture for satellite refueling tasks, but the architecture could be extended to other applications, such as in-space servicing, assembly and maintenance, and to support multiple operators and robots.

## 2 Materials and Methods

**FIGURE 1:**
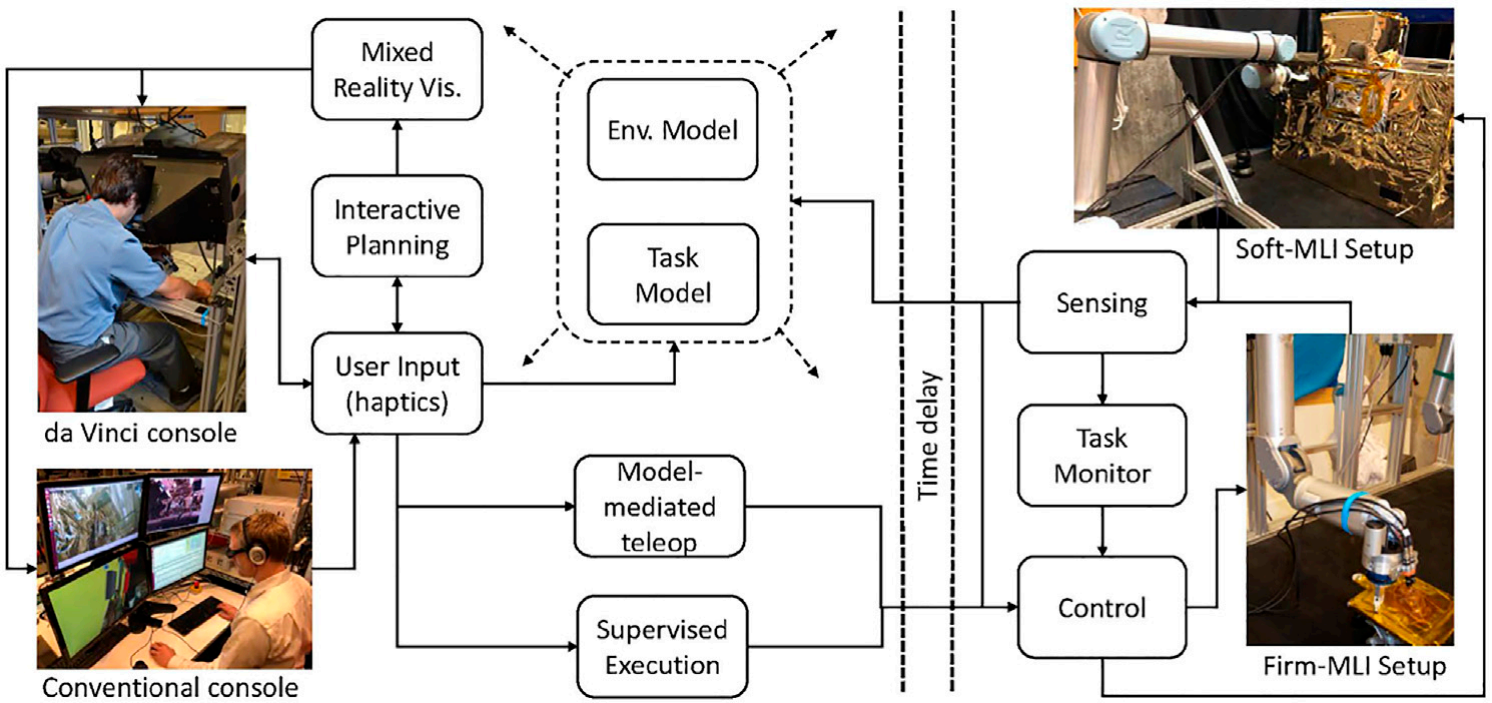
Model-based architecture, showing two teleoperation consoles (conventional and da Vinci) and two test platforms (Firm-MLI and Soft-MLI setups).

Figure 1 shows an overview of our model-based architecture. The models are created based on sensor feedback from the remote environment and/or by operator input. Although not explicitly shown in the figure, the models are used by all other system components. In addition to the conventional direct teleoperation, we created two primary control approaches: model-mediated teleoperation and interactive planning with supervised execution (IPSE). In model-mediated teleoperation, the operator works with a simulated model and the results of the simulation are streamed into space. The robot in space uses sensor-based control to attempt to recreate the simulation, and contains a task monitor to detect when it has failed. In the IPSE system, the operator plans robot motions in the simulated environment, with the ability to preview and adjust the motions before sending them to the robot.

Figure 1 also shows the two teleoperation consoles used in this research:

1) **da Vinci master console:** This consists of mechanical components from a da Vinci Surgical System (Intuitive Surgical, Inc., Sunnyvale, CA), coupled with the open-source electronics and software provided by the da Vinci Research Kit (dVRK), Kazanzides et al. (2014). The master console contains two 7-DOF Master Tool Manipulators (MTMs) for user input and haptic feedback, a stereoscopic display, and footpedals.
2) **Conventional monitors and keyboard:** The conventional console consists of multiple monitors, keyboard and mouse. In some experiments, we add a 3D mouse and/or a 3D monitor with shutter glasses.

**FIGURE 2:**
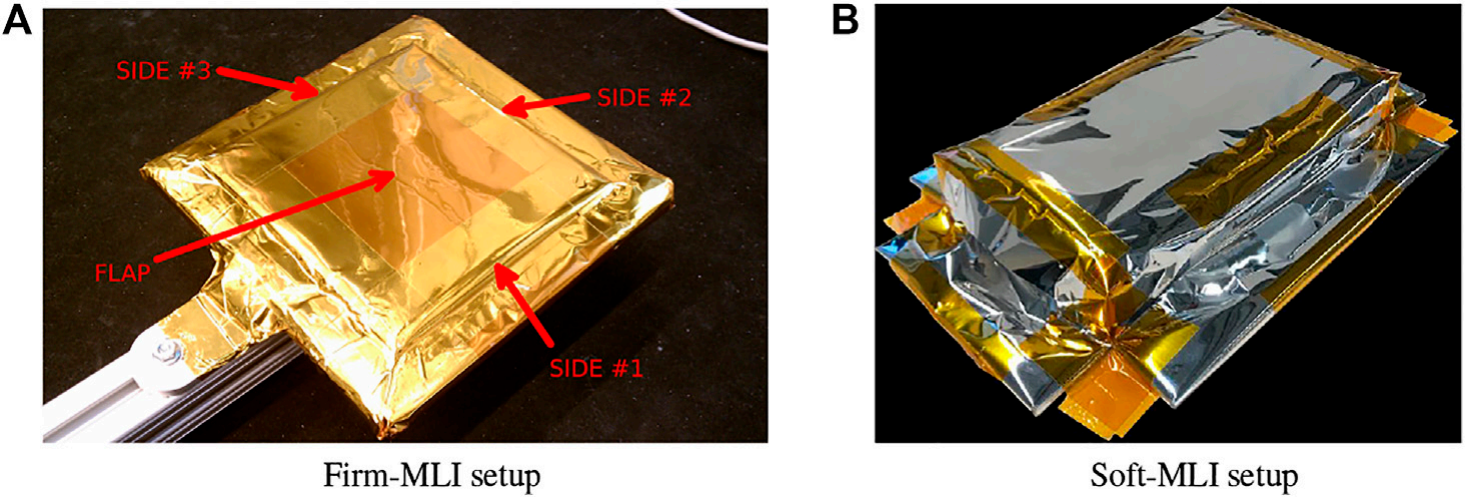
Test platforms: **(A)** Firm-MLI setup, with MLI flap taped onto rigid surface of underlying satellite; **(B)** Soft-MLI setup, with MLI in shape of a rectangular hat.

In addition, Figure 1 shows the two ground-based test platforms used for the experiments. The Firm-MLI setup consists of a 7-DOF Whole Arm Manipulator (WAM) robot (Barrett Technology, Boston, MA) equipped with a stereo tool camera and a wrist-mounted force sensor (JR3, Inc., Woodland, CA). The primary workpiece is an aluminum plate with an MLI flap, as shown in Figure 2A. The Soft-MLI setup consists of a UR-5 or UR-10 robot (Universal Robots, Odense, Denmark), with the same JR3 wrist-mounted force sensor, a monocular tool camera (PointGrey BlackFly, FLIR Integrated Imaging Solutions, BC, Canada), and a rotary cutting motor. The workpiece is a mock satellite that includes a soft MLI hat structure (Figure 2B), beneath which are three thin-walled stainless-steel tubes to emulate fill/drain ports.

The following sections provide further details on the major components of the architecture. For clarity, we separate the initial model construction from the model update.

### 2.1 Model Construction

Figure 1 indicates that models can be created from sensor data or via user specification. The following sections present an example of each: Section 2.1.1 describes how sensor (camera) data can be used (with manual feature identification) to create an environment model, and Section 2.1.2 indicates how operator input can be used to create a task model.

#### 2.1.1 Creating an Environment Model

**FIGURE 3:**
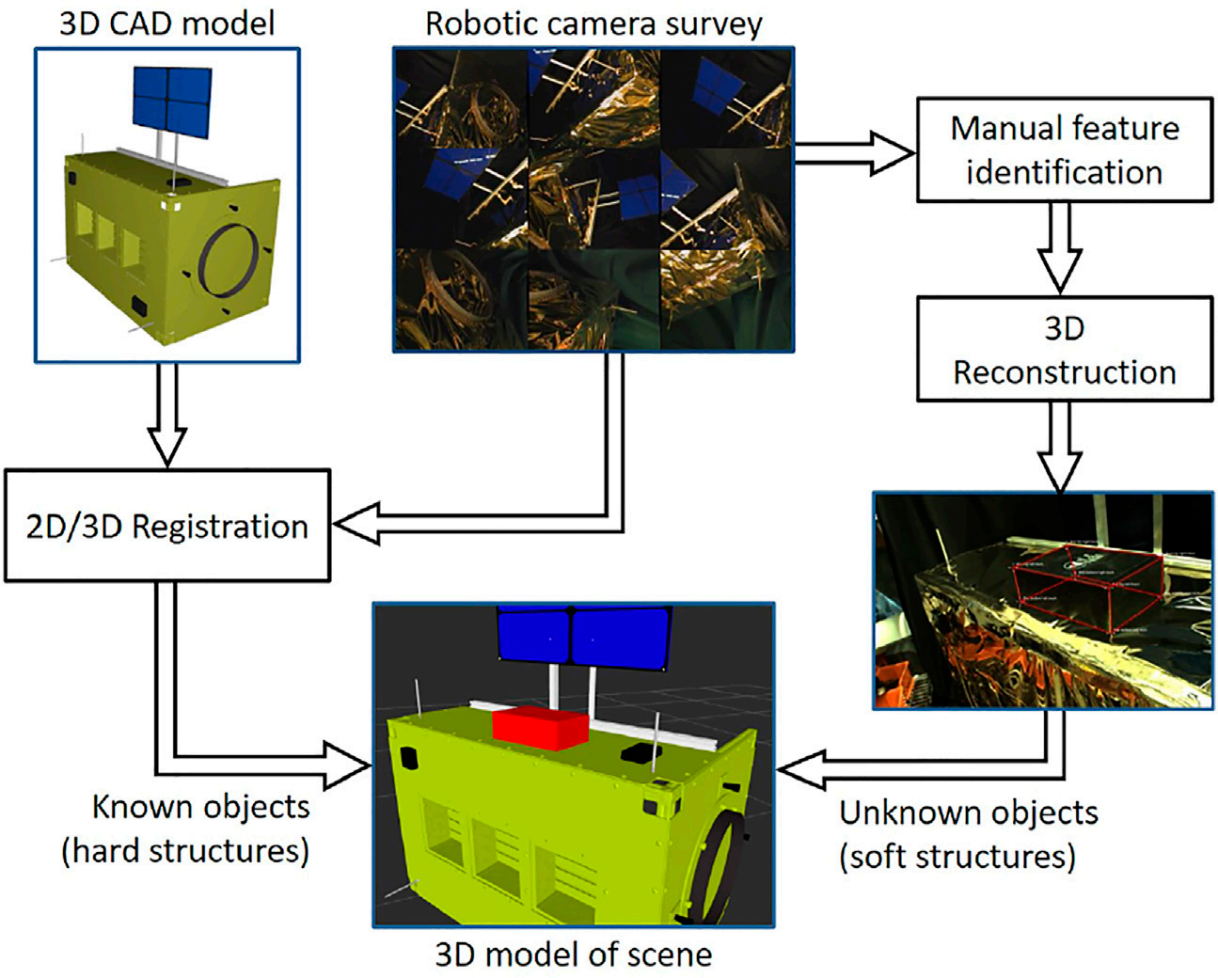
Creating an environment model from a robotic 2D image survey by registering to known objects (satellite) and reconstructing unknown or imprecisely known objects (MLI hat).

During teleoperation, the operator often views the remote environment *via* one or more camera images. In these cases, there is no environment model other than the mental model in the operator’s imagination. For improved visualization of the remote environment, however, it is possible to create a 3D environment model from multiple 2D camera images acquired during a robotic survey. The process consists of registration to known objects and 3D reconstruction of unknown (or imprecisely known) objects, as shown in Figure 3.

*Registration to known objects:* We register to known objects, such as a satellite, by locating the object’s natural landmarks within the images, then using pose estimation to find the object pose that best fits these observations. If the camera’s pose (extrinsic parameters) is known from robot kinematics, then the object’s pose with respect to the camera will also yield a registration of the satellite to the robot’s base frame. Pose estimation is sensitive to the landmark observation accuracy; thus, we combine pose estimates from multiple camera viewpoints to obtain more accurate registration.

This registration procedure requires the camera’s extrinsic and intrinsic parameters. The camera intrinsics can be calibrated prior to launch, and they are unlikely to change during the mission. However, it is possible to re-calibrate the camera during flight using a checkerboard pattern or natural landmarks. Similarly, the extrinsic parameters of the tool camera can be calculated using either natural features or a checkerboard pattern. For this hand-eye calibration, we first use the method of Tsai and Lenz (1988) to solve the conventional *AX* = *XB* hand-eye formulation, then refine *X* using reprojection error minimization.

*Reconstruction of unknown objects:* Unknown (or imprecisely known) objects, such as the MLI hat on the Soft-MLI setup, are reconstructed by manually locating natural landmarks on the object that are unambiguously identifiable on at least two images taken from different view angles. Once the landmark observations are added, the system can automatically calculate the landmark positions in 3D space with respect to the world coordinate frame. The triangulation problem can be solved using a closed-form least squares method to find the best positions given at least two observations per landmark. Knowing the 3D coordinates of the landmarks enables the user to create triangular or quadrilateral ‘faces’ between the landmarks and build a model of the unknown object. The landmarks serve as vertices and the faces are converted into triangles that form the topology of the mesh.

#### 2.1.2 Creating a Task Model

The goal of teleoperation is to achieve a task objective. An operator may attempt to accomplish the task by using an input device, such as a joystick or keyboard, to issue motion commands to the remote robot. By creating a task model, the operator can “configure” the teleoperation system to provide assistance to complete the task. One common example is a virtual fixture, Rosenberg (1993), which performs a function analogous to that of a physical fixture (e.g., a ruler) and can be adjusted in a virtual environment at run time. Virtual fixture primitives, such as “stay above a plane”, “move along a line”, and “rotate about a line”, can be combined to provide assistance for complex manipulation tasks, Kapoor et al. (2006).

**FIGURE 4:**
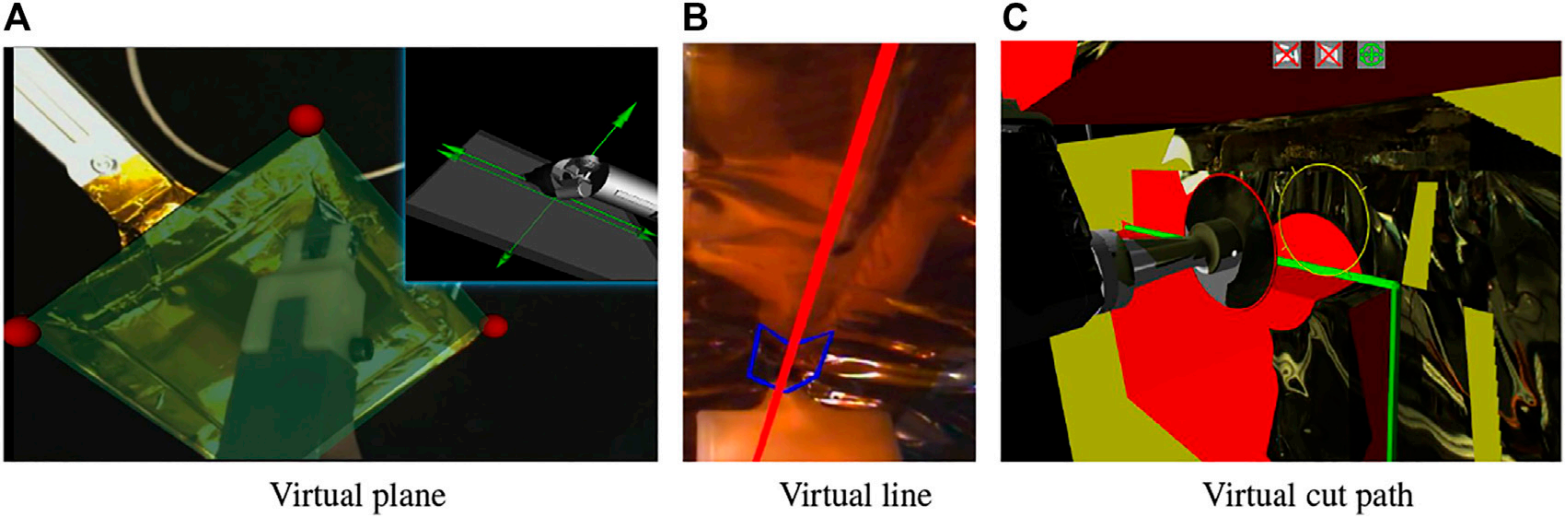
Virtual Fixtures: **(A)** Virtual plane on (stereo) camera image, Xia et al. (2012); **(B)** Virtual line (red) on (stereo) camera image; **(C)** Virtual cut path (green lines) on 3D environment model, Pryor et al. (2019).

Xia et al. (2012) described a user interface that enables the operator to place graphical primitives, such as planes and lines, to define task goals and/or constraints. This task model can be transformed into virtual fixtures for haptic feedback to the operator and can define frames for hybrid position/force control on the remote robot, Xia et al. (2013). This requires a registration between the remote environment and the operator’s environment, which can be a virtual environment created by modeling the remote environment (Section 2.1.1), or can be real images of the remote environment. In the latter case, the graphical primitives are implemented as augmented reality overlays on the real images. Figure 4 shows examples of these cases: (A) placing a virtual plane, on the camera image, to push against while sliding along to cut the tape in the Firm-MLI setup, Xia et al. (2012), (B) overlaying a virtual line on the camera image to guide cutting, and (C) overlaying the cut path (green lines) on the virtual model for cutting the MLI hat in the Soft-MLI setup, Pryor et al. (2019).

The first two examples illustrate placing virtual objects on stereo camera images, which were displayed on the da Vinci master console shown in Figure 1. In general, it can be difficult to visually align virtual overlays with real objects, especially in depth, but this is not an issue in these examples. The first example, placing a virtual plane, causes the remote robot to use a hybrid position/force controller, Raibert and Craig (1981), where force control is used in the direction of the plane normal. Thus, the method is not significantly affected by errors in the plane depth or angle. Nevertheless, we developed two methods to address this concern. First, we developed a method to enable an operator to more accurately align the virtual plane by dynamically texturing the virtual plane with the purpose of adding visual distortion until the virtual plane is accurately aligned with the real plane, Leonard (2015). Then, Li and Kazanzides (2016) reported the development of a method to update the orientation of the virtual plane based on sensor feedback during the task, as described in Section 2.5.

The second example, placing a virtual line (Figure 4B), is a virtual fixture that is created by the operator during the task and is thus already aligned with the camera image. In particular, the operator disengages teleoperation and uses the roll joint of the MTM to define the orientation of a virtual fixture line that passes through the tool center and is in the plane defined by the cutting tool, Chen (2017). When the operator resumes teleoperation, motion of the robot will be confined to the virtual fixture line. Additional virtual fixtures, including a nonholonomic virtual fixture, are described in Section 2.3.1.

In the third example (Figure 4C), the virtual fixture is defined in the 3D environment model and is therefore already registered with the remote robot.

### 2.2 Mixed Reality Visualization

Visualization is critical for the operator’s situation awareness and ability to telerobotically interact with the remote environment. However, the typical visualization approach is to view the delayed video feedback from one or more cameras. These cameras may not be optimally placed for a specific task and, in many cases, the best view is provided by a camera mounted on the robot end-effector. Teleoperation with a tool-mounted camera (i.e., an “eye in hand” configuration) is not intuitive and its proximity to the end-effector generally results in a limited field-of-view.

Mixed reality can be used to address some of these visualization issues. The two most recognized mixed reality concepts are augmented reality (AR) and augmented virtuality (AV), Milgram and Kishino (1994). Both combine visual representations of real and virtual environments. In AR, virtual objects are overlaid on video streams. Since the 1990s, NASA has been experimenting with AR in teleoperation while servicing the ISS and other satellites to improve the operators’ situational awareness, Ince et al. (1991).

In contrast, in augmented virtuality (AV) the result is a computer generated rendering of the environment in which registered real-life images are overlaid on virtual objects. This approach enables visualization from arbitrary points of view, as opposed to AR, where the location of the camera is fixed. AV also enables the rendering of stereoscopic views of the scene, which has been shown to improve teleoperation performance, Spain (1991).

The following sections describe some implementations of augmented reality and augmented virtuality. We do not include virtual reality (VR), where the entire scene is based on the models (i.e., no reality), but this is used for the interactive planning module described in Section 2.3.2.

#### 2.2.1 Augmented Reality

Augmented reality (AR) is often used for systems where the primary visualization is the camera image. In a conventional teleoperation console, such as shown in Figure 1, there are many displays available for presenting information to the operator; thus, it is generally not necessary to obstruct the camera image to display additional information. Where AR is useful, however, is to overlay virtual objects that are registered to the camera image. Perhaps the best example is commanded or predictive display, Bejczy and Kim (1990); Lane et al. (2001), which attempts to show the position of the robot without the effects of time delay. In commanded display, the current commanded position (e.g., based on the operator’s command *via* keyboard, joystick or haptic device) is used to position a virtual overlay of some part of the robot, often just the end-effector, on the image. Examples of commanded display are the blue chevron in Figure 4B, which shows the commanded position of the chevron-shaped cutting blade, and the red ring in Figure 4C, which shows the commanded position of the rotary cutter (in this figure, the robot is not moving so the red ring is aligned with the cutter image). Note that Figure 4C also shows a yellow ring, which is similar to commanded display but indicates the contemplated pose of the circular cutting blade. When using the keyboard interface, the operator can enter a robot position into a text box, which updates the pose of the yellow ring but does not cause robot motion unless the operator presses a “move” button. Technically, predictive display shows the position of the robot (and possibly changes to the environment) based on a simulation of the commands being sent to the robot. However, in many cases, the implementation of predictive display is the same as commanded display and the terms are used interchangeably.

For cases where a task model is defined, it can also be helpful to overlay graphical primitives associated with that task model. Figure 4A shows an example of a virtual plane and Figures 4B,C show examples of a cutting path.

#### 2.2.2 Augmented Virtuality

We developed an augmented virtuality visualization where the operator primarily visualizes the 3D model of the scene, which can be presented in stereo and from any perspective, Vagvolgyi et al. (2017, 2018). This model is augmented by projections of the live (delayed) video onto the 3D model.

Initially, we implemented the augmented virtuality visualization in RViz, the visualization package provided with ROS, Kam et al. (2015). RViz does not provide dynamic texturing features by default, but custom RViz plugins may access lower level capabilities provided by the underlying Ogre 3D (Open source GRaphics Engine) package, which provides an object-oriented abstraction to low-level GPU hardware features. We therefore created a texture projection plugin which adds a second step to the RViz rendering loop to render the scene from the point of view of the projector (real camera) and assign new values to the textures based on the image projection. As a result, a second textured material layer is added to the 3D model that mimics the appearance of an image being projected on the model. However, we discovered several limitations with this approach: images were also projected onto rear surfaces, occlusion (shadowing) was not handled, and it was unsuitable for mosaicking or image blending (e.g., of multiple camera images). We developed some workarounds, such as creating an image mask to prevent the cutter image from being projected onto the satellite surface (due to lack of shadowing). But, we also discovered performance limitations, due to insufficient low-level access and the fact that the GPU must render the image twice.

Although it may be possible to resolve these feature and performance limitations through more elaborate customization of Ogre 3D, we chose to implement a new renderer in C++, using OpenGL, as reported in Pryor et al. (2019). The new renderer performs real-time ray-tracing to project the camera images with correct occlusions on the 3D scene, thereby mapping the image of the tool assembly on the tool model and the image of the satellite on the satellite model, without the need of an image mask. The 3D models in the scene are all wrapped in high resolution texture, and the renderer is capable of adding multiple camera projections to the texture using mosaicking techniques to cover the visible parts of the satellite model with registered real-life camera images. On top of the static mosaic, the system also maps on the scene the time-delayed video streams captured from the cameras. All this is performed real-time, enabling a more realistic and dynamic 3D visualization. The new renderer also enables the display of a variety of status indicators in the 3D view. The indicators are rendered as icons and text overlays (see icons at top of Figure 4C). In this figure, the 3D model includes the satellite CAD model (yellow), reconstructed MLI hat (red), and robotic tool. The robot model is updated by the delayed telemetry from the remote robot. As in the augmented reality display, overlays include the commanded robot position (red ring), the contemplated robot position (yellow ring), and the desired cut path (green lines).

### 2.3 Semi-Autonomous Teleoperation

While predictive display, Bejczy and Kim (1990), was experimentally shown to help operators perform positioning tasks, it (and the related predictive control method) are not feasible when the robot must contact the environment because current models cannot accurately predict the future state of the system. Bilateral teleoperation reflects the sensed environment force back to the operator (e.g., *via* haptic feedback), but has been shown to be impractical and unstable under small to medium delays, Hashtrudi-Zaad and Salcudean (2000). Wave variable encoding of the force and motion can restore control stability, Niemeyer and Slotine (2004), but delayed force feedback has not proven to be intuitive to human operators. Thus, many researchers have focused on semi-autonomous teleoperation. This includes supervisory control, Sheridan (1993), where the operator issues high-level goal specifications that are autonomously executed by the remote robot, and model-based methods, such as teleprogramming, Funda et al. (1992); Sayers et al. (1998), tele-sensor-programming, Hirzinger et al. (1993), and model-mediated teleoperation, Mitra and Niemeyer (2008), where the operator interacts with a model (simulation) of the remote environment and the results of that interaction direct the motions of the remote robot. At a high level, the model-based methods are similar because they all create a model from some combination of á priori knowledge and remote sensor feedback. Teleprogramming and tele-sensor-programming focus on creating the model on the master side, using á priori task knowledge and possibly also an initial survey of the remote environment, Funda et al. (1992), whereas model-mediated telemanipulation focuses more on the sensor-based model update, for example, to explore a mostly unknown environment. Supervisory control implicitly assumes that the operator has a model to determine the sequence of goal specifications.

The following sections present two semi-autonomous teleoperation approaches implemented within the proposed architecture: model-mediated teleoperation and supervisory control.

#### 2.3.1 Model-Mediated Teleoperation

The preferred cutting strategy in the Firm-MLI setup (Figure 2A) is for the cutting blade to puncture the tape seam, then press down against the satellite surface while sliding along to cut the seam. Xia et al. (2012) initially defined a task model that consisted of a plane to represent the satellite surface, as discussed in Section 2.1.2. For the model-mediated approach, once the virtual plane was defined, it provided haptic feedback to the operator and prevented the operator from moving beyond the plane or from changing the orientation of the cutter with respect to the plane normal (rotation within the plane; i.e., around the plane normal, may be permitted). On the remote robot, the user-specified plane determined the task frame for hybrid position/force control; specifically, the plane normal defined the direction for force control. Thus, while the operator interacted with the simulated environment, the remote robot used sensor-based control to attempt to reproduce this simulation, Xia et al. (2013).

Xia et al. (2013) used a constrained optimization controller, Kapoor et al. (2006), to implement the virtual fixture on the master. In particular, the controller computed an optimal incremental motion Δ**x**_*m*_ based on the operator’s desired incremental motion $\Delta x_{m}^{d}$ by solving a constrained optimization problem of the form:

$$\begin{array}{l} \underset{\Delta x_{m}}{min}\;{\left\|\begin{array}{c} \Delta x_{m}-\Delta x_{m}^{d} \end{array}\right\|}^{2} \end{array}$$ (1)

$$\begin{array}{l} s.t.\;\begin{array}{c} h_{1}\left(\Delta x_{m}\right)<0\\ \vdots \\ h_{N}\left(\Delta x_{m}\right)<0 \end{array} \end{array}$$

where *h*_1_, … , *h*_*N*_ represent the constraints of *N* virtual fixtures.

The task model was subsequently enhanced to include various forms of line constraints to assist with the cutting task. Xia et al. (2012) had enabled the operator to graphically define a line, corresponding to the tape seam on the camera image. However, while a line virtual fixture would seem to be the most appropriate for cutting along a straight edge, it can sometimes be too constraining because the virtual line must be correctly registered to the actual tape seam and because cutting anomalies such as bunching or tearing of the tape may require the operator to stray from the line. Chen (2017) presented one solution, which was to enable the operator to adjust the line virtual fixture during the task, using the roll axis of the da Vinci MTM to set the line orientation, as shown in Figure 4B.

As an alternative, Vozar et al. (2015b) developed a (software-imposed) virtual nonholonomic constraint (VNHC), motivated by the hypothesis that the difficulty of commanding three degrees of freedom (on the plane) could potentially be mitigated by reducing the number of inputs to the system, particularly because there is no requirement to move instantaneously in a lateral direction. By selecting a familiar nonholonomic constraint, such as one similar to driving a car, a natural mapping from input to output space can be achieved.

The VNHC was based on a unicycle (also referred to as a rolling wheel), as it is simple, intuitive, and the steering angle can be controlled independently from the planar position. The constraints for a unicycle are given by Spong et al. (2006):

$$\begin{array}{cc} \dot{x}-r\dot{\phi }\cos\theta & =0\\ \dot{y}-r\dot{\phi }\sin\theta & =0 \end{array}$$ (2)

where *x* and *y* are the Cartesian position of the center of the wheel, *θ* is the heading angle, *r* is the radius of the wheel, and $\dot{\phi }$ is the angular rotatational velocity of the wheel. The forward speed of the wheel can be given as $v=r\dot{\phi }$ . This constraint was applied directly to the planar model of the cutting blade to impose the unicycle constraint on the end effector. The motion of the cutter was then controlled directly by the operator, who provided the desired velocity, $v={\dot{x}}_{m}$ , and steering angle, *θ* = *θ*_*m*_, via an input device.

The VNHC can be implemented without specification of a desired line constraint. However, it is also possible to incorporate a soft virtual fixture in the nonholonomic formulation, which guides the operator toward the virtual fixture line, but allows motions away from the line with increased effort. In particular, Vozar et al. (2015b) introduced a PD controller that determined a cutter angle (*θ*_*PD*_) based on the lateral error (*y*_*err*_ = *y* − *y*_*VF*_) of the cutter, where the virtual fixture line is in the *x* direction with a lateral offset of *y*_*VF*_:

$$\theta_{PD}=K_{p}y_{err}+K_{d}{\dot{y}}_{err}$$ (3)

The motion of the cutter was then controlled from position commands *x*_*m*_ and *θ*_*m*_ as:

$$\begin{array}{cc} v & ={\dot{x}}_{m}\\ \theta & =\theta_{m}+{\dot{x}}_{m}\theta_{PD} \end{array}$$ (4)

Note that with no angular input from the operator, the cutter follows the PD controller’s inputs to orient and align with the virtual fixture. The operator is able to override the cutting angle from the PD controller with the input *θ*_*m*_, thus making this a soft virtual fixture.

#### 2.3.2 Interactive Planning and Supervised Execution (IPSE)

**FIGURE 5:**
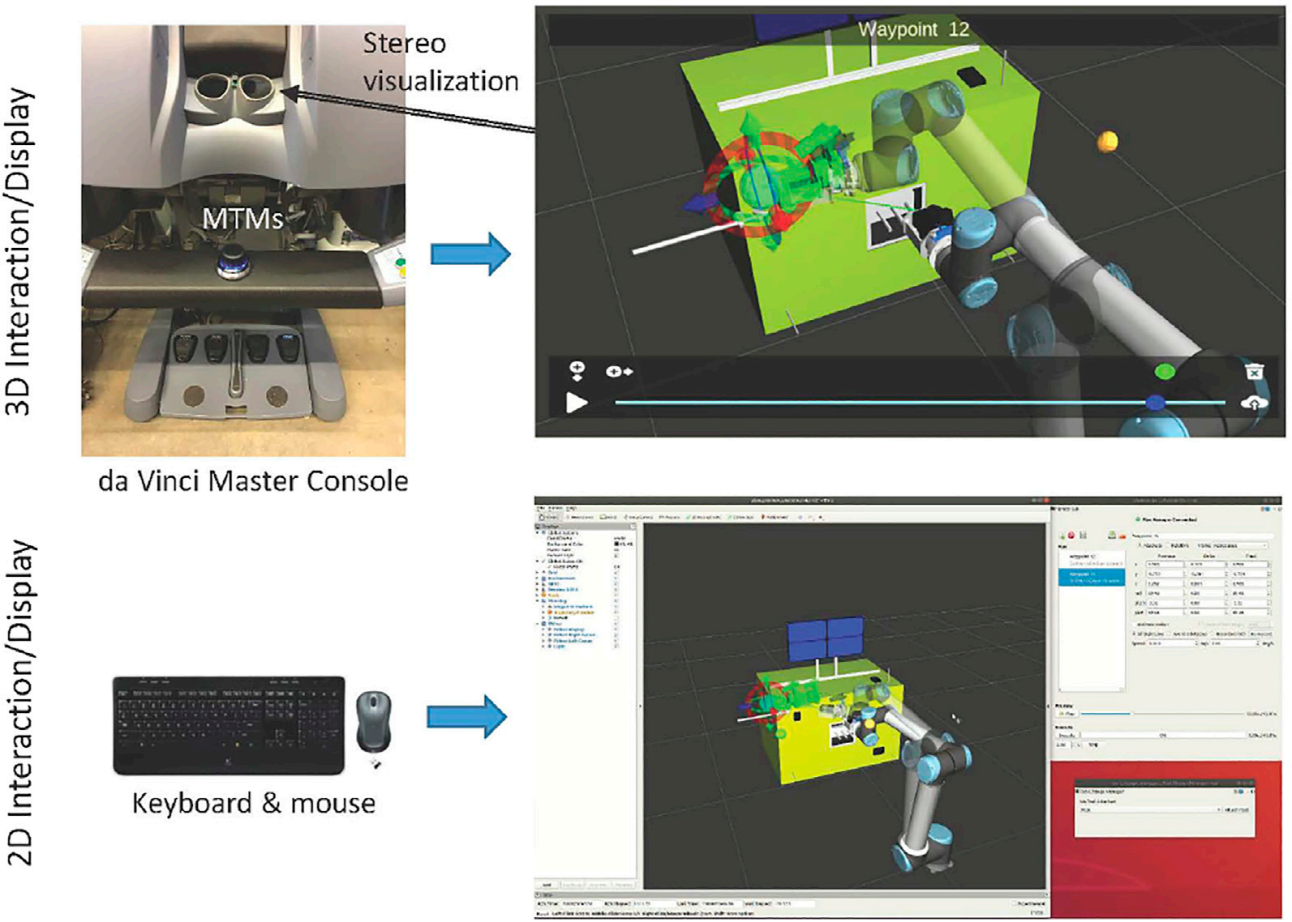
Overview of Interactive Planning System, showing 3D interface **(top)** and 2D interface **(bottom)**. The da Vinci Master Console includes two Master Tool Manipulators (MTMs) that enable 3D interaction (for example, to move the interactive cursors) and provides a stereo display for visualization. Both 3D and 2D interfaces are available simultaneously and visualize/update the same scene.

The IPSE module, Pryor et al. (2019), shown in Figure 5, relies on the 3D environment model described in Section 2.1.1 and is independent from any user interface. It communicates with any number of interfaces simultaneously and changes made in any interface are immediately reflected in all connected interfaces. We implemented two user interfaces, as shown in Figure 5: a 2D mouse-and-keyboard interface composed of a custom GUI with RViz for visualizations and a 3D interface operated with the master console of a da Vinci surgical robot.

Related work, outside the domain of space robotics, includes the use of mixed/augmented reality for visual programming of robot motions. In Quintero et al. (2018), a user can plan paths as a series of waypoints in an augmented reality (AR) environment, preview and edit the paths, and then execute them either autonomously or by allowing the user to control progress through the path. In Gadre et al. (2019), the user similarly builds a path out of primitives, visualizes the final path, and then executes it.

Within the IPSE environment, the operator creates a motion plan using the interactive planning capability, previews the resulting robot motion and edits the plan if necessary, and then executes the plan with supervised execution. These steps are repeated until the task is complete. This is essentially an implementation of high-level supervisory control originally articulated by Sheridan (1992, 1993).

A motion plan consists of a series of waypoints, where each waypoint represents an intermediate destination in the motion plan. A motion planning engine, using the MoveIt planning framework, Chitta et al. (2012), plans a trajectory to connect each waypoint’s destination pose with the final configuration of the previous waypoint’s trajectory, with the first waypoint connected to the robot’s current configuration. The resulting trajectories are collision-free when possible and marked as invalid when a collision cannot be avoided.

The operator may configure each waypoint to use a straight-line path, which causes the end effector to follow a straight line in task space; to avoid obstacles, in which case the motion planner may select any collision-free path; or to follow the same task-space path that the operator followed to move the waypoint marker. Each waypoint trajectory also has an independent set of desired speeds, both linear and rotational.

When the operator is satisfied with the planned trajectory, they may execute the entire trajectory on the remote robot, or choose to “Step” through a trajectory to monitor it more closely. The step function truncates the trajectory to the specified time and sends only the truncated portion to the robot. During execution, the operator may observe the robot’s progress in an Augmented Virtuality (AV) visualization environment (Section 2.2), where the robot and environment models are augmented with a projection of the image from the robot tool camera. The projection improves the operators’ situational awareness and ability to judge the completion of the task by transforming the 2D image into 3D textured objects; furthermore, it helps operators recognize inconsistencies between the model and reality.

At present, the IPSE framework only supports position-based trajectories, but it would be possible to implement other types of motion primitives, such as compliant motion. These motions, however, are challenging to preview because they would require environment models with accurate geometry and material properties, as well as realistic dynamic simulation, whereas the current IPSE framework only requires accurate geometric models and kinematic simulation of the robot.

### 2.4 Model-Based Monitoring

The availability of models enables monitoring of tasks in the remote environment. If the monitoring can be implemented on the remote system (within the computational constraints of available hardware), it has the advantage of being able to immediately react to failure, without having to wait for the operator to recognize the problem in the delayed video feedback and provide corrective action via time-delayed control. Alternatively, if the monitoring is implemented on the ground-based system, it is subject to telemetry delay, but could potentially detect and react to failures more quickly than the human operator. This section presents two examples of task monitors that both detect cutting anomalies, but using different sensors.

#### 2.4.1 Force-Based Monitoring of Cutting

For compliant motion implemented by hybrid position/force control, as described in Section 2.3.1, the system controls position (or velocity) in certain directions of the task frame and controls force in the others. In the directions of position/velocity control, it is feasible to measure the force and, given an adequate model of the task and environment, to implement a model-based task monitor.

**FIGURE 6:**
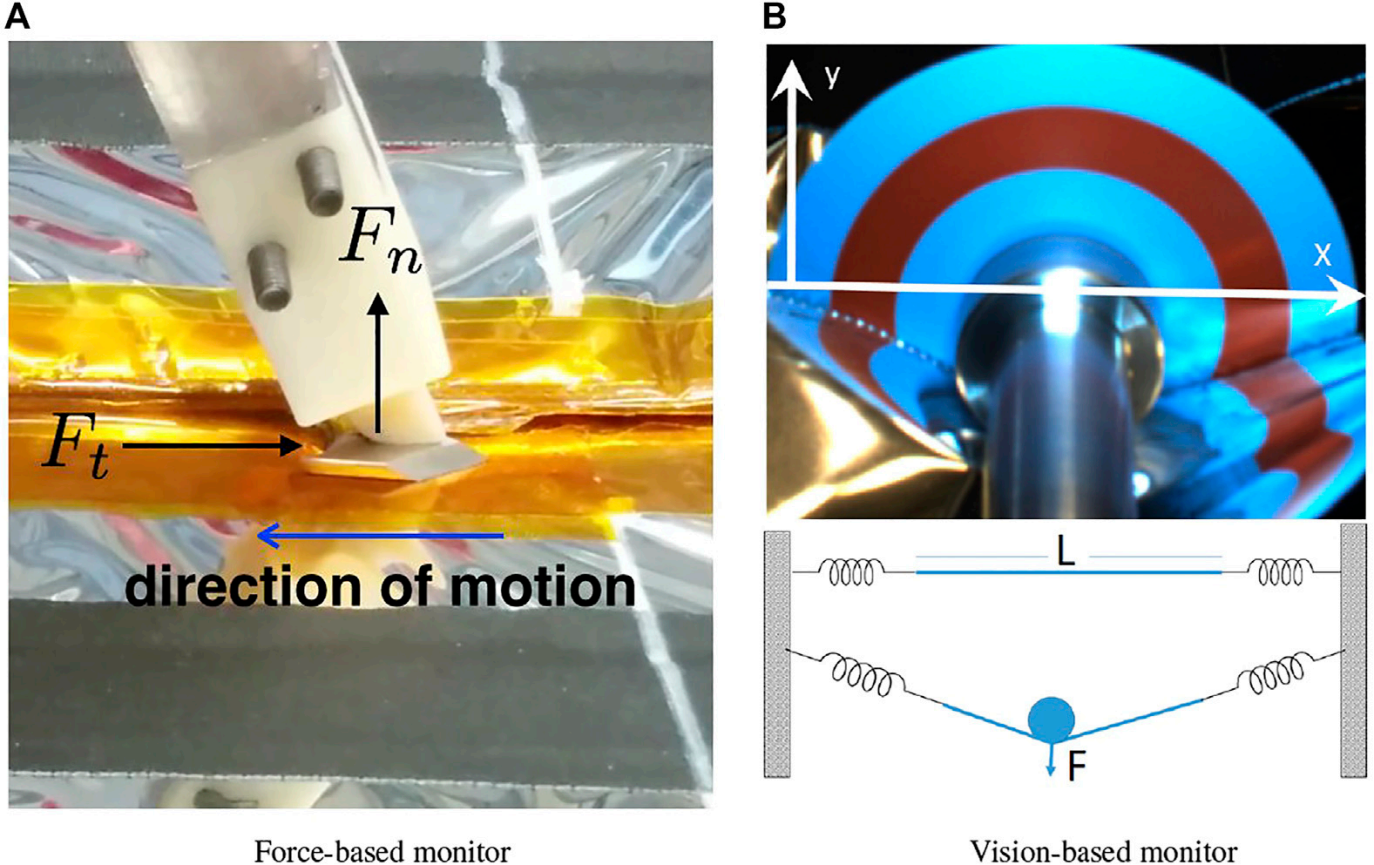
Two strategies for monitoring cutting performance: **(A)** force-based and **(B)** vision-based, with 2D sketch of model on bottom.

As an example, consider the Firm-MLI setup shown in Figure 6A, where the cutting blade pushes against the surface and slides along the tape seam (compression-based strategy). Kandaswamy et al. (2014) and Xia et al. (2013) determined that a large component of the force in the direction of cutting is due to the friction between the cutter and tape surface, which can accurately be modeled as kinetic friction. The kinetic friction was estimated by sliding the cutter along the tape (while not cutting). The force due to cutting the tape was adequately modeled as a constant, which was determined by measuring the force while cutting and then subtracting the previously measured frictional force. The final model is:

$${\hat{F}}_{t}=\mu_{k}\left|F_{n}\right|+F_{c},$$ (5)

where ${\hat{F}}_{t}$ is the expected force in the direction of cutting, *F*_*n*_ is the measured normal force, *μ*_*k*_ is the experimentally-determined coefficient of kinetic friction (0.56 in these experiments) and *F*_*c*_ is the experimentally-determined cutting force (approximately 4N). This simple model should be feasible to evaluate even with the limited computational resources available in space. The concept is that the on-orbit robot system would use the model to estimate the expected force in the direction of cutting, ${\hat{F}}_{t}$ , and stop motion if the measured force, *F*_*t*_, is significantly higher or lower. In particular, Kandaswamy et al. (2014) showed that many cutting anomalies could be detected by checking whether the measured force was outside a threshold of 30% above or below the estimated force. See also Section 2.5.2, which introduces an adaptive estimator to update the model parameters *μ*_*k*_ and *F*_*c*_.

#### 2.4.2 Vision-Based Monitoring of Cutting

For the Soft-MLI setup, a rotating cutting blade is used to cut the MLI hat, rather than the fixed cutting blade employed in the Firm-MLI setup. In consequence, cutting forces are dramatically reduced, and cutting the MLI hat will not produce significant force measurements. Mahmood et al. (2020) proposed a model that estimates the applied force on the MLI from visual feedback from the tool-mounted camera, as shown in the 2D illustration of Figure 6B. The surface of the hat was represented by a string of length *L* attached at both ends by two springs. As the shaft of the blade pushes on the string, it exerts a force *F* that pushes the string downward. This interaction between the string and the shaft creates two angles *α*_1_ and *α*_2_ on either side of the shaft and the relation between the applied force and the angles is *F* = *F*_1_  cos (*α*_1_) + *F*_2_  cos (*α*_2_) where *F*_1_ and *F*_2_ are determined by the spring coefficients and their displacements. Mahmood et al. (2020) proposed to use visual feedback to assess this force because cameras are available to the operators. Although the blade applies little force to the MLI, the shaft that holds the blade applies the bulk of the force (albeit a small one) as it pushes on the surface to make sure that the blade cuts through all the layers of the hat.

This force can be observed visually as the MLI passes under the shaft, as seen in Figure 6B, and the more the shaft pushes down, the more a “V” shape is observed on each side.

It is, however, a challenging computer vision problem to detect the interface between the MLI and cutting blade due to the presence of metallic reflective film (kapton or aluminum). To compute the angle robustly, the implementation used concentric circles with colors that offer a sharp contrast in a color space (e.g., the red ring visible in Figure 6B). In particular, the HSV color space was selected and the red and cyan colors of the H (hue) channel were used. These colors are 180° apart on the hue channel which ranges between 0 and 360°. Canny edges are extracted from the hue channel and the result is masked with predefined templates of two concentric thin rings where the transition between colors is expected. The expected result is a long edge on each side of the red circle from which the endpoints are found. These endpoints represent the coordinates where the MLI occludes the blade by altering the expected hue pattern. By fitting a line through each pair of endpoints on both sides of the shaft, the angles described by the MLI on both sides of the shaft are computed. The angles quantify the engagement of the cutter and MLI, which is proportional to the applied force and is sufficient to monitor whether the cutter is too shallow or too deep. It could also potentially be used in a hybrid control scheme where the operator controls motion along the cutting path and the system automatically controls the engagement depth. Determination of actual force values would require estimation of parameters such as the stiffness of the MLI, which can be a topic of future research.

### 2.5 Model Update

While a static model may be sufficient for some tasks, it is often necessary to update the environment or task model during operation. This section presents two examples of model updates. The first example updates the alignment of the task model (a virtual plane) with the real environment (satellite surface), in the model-mediated teleoperation approach first presented in Section 2.3.1. This could alternatively be viewed as updating an environment model, with the virtual plane representing the satellite surface. The second example updates parameters of the task model used to monitor the cutting force in Section 2.4.1.

#### 2.5.1 Correcting Task Frame Misalignment

**FIGURE 7:**
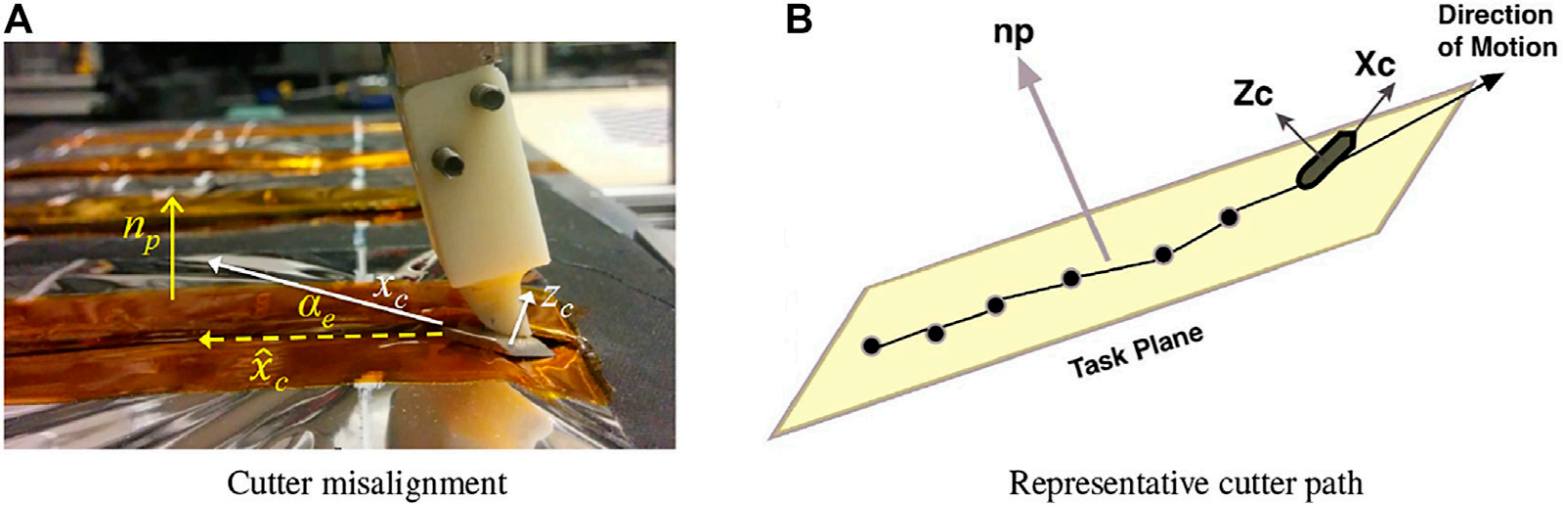
Correcting task frame misalignment. **(A)** definition of terms: cutter axis *z*_*c*_ is not perfectly aligned with plane normal *n*_*p*_, leading to misalignment between commanded cutter direction *x*_*c*_ and actual cutter direction ${\hat{x}}_{c}$ resulting from hybrid position/force control; **(B)** representative illustration of a cutter path on the plane.

Section 2.3.1 presented a model-mediated teleoperation implementation where the remote robot used a hybrid position/force controller to allow motion along a plane while controlling force normal to the plane. Figure 7A shows the configuration of the task. The cutter axis *z*_*c*_ should be aligned with the plane normal *n*_*p*_, but registration error between the virtual plane model and the physical satellite surface will cause misalignment. A large misalignment can significantly reduce the task quality and is likely to cause adverse events such as the cutter digging into the access panel, potentially damaging both the robot and the satellite. This provides the incentive to estimate misorientation during cutting and update the task model. In Section 2.4.1, we observed that in the directions of position/velocity control, the measured force could be used to monitor the task performance. Here, we consider that in directions of force control, the measured position can be used to update the task model, Li and Kazanzides (2016).

The goal of the technique proposed by Li and Kazanzides (2016) is to allow the remote robot to automatically align the cutter to the plane, during the cutting task, by estimating the two DOF rotation between *z*_*c*_ and *n*_*p*_. The problem is illustrated in Figure 7B. The yellow plane indicates the satellite access panel and the solid line is the cutter path, with black dots representing positions along the path. The goal is to use this position information along with measurements from the force sensor to perform online estimates of the plane normal *n*_*p*_. Because the cutter normal *z*_*c*_ is known, alignment error can be calculated and corrected.

The use of position and/or force measurements to adjust the task frame for hybrid position/force control is well studied; some early studies include Merlet (1987); Kazanzides et al. (1989); Yoshikawa and Sudou (1993). These studies focused on estimating a local task frame for a robot in contact with an unknown, or partially-known, object. A similar problem was studied by Karayiannidis and Doulgeri (2006), who also assumed compliant contact with a plane and developed an adaptive controller to estimate the plane normal.

This implementation differed from prior work due to the requirement to address two application-specific challenges. The first challenge, also considered by Karayiannidis and Doulgeri (2006), was due to the compliance of the MLI blanket covering. If the normal force varied during the cutting process, the sampled points would have varying offsets with respect to the underlying plate and thus would not allow an accurate estimate of the plane orientation. The second issue arose due to the task objective, where the goal was to cut along the seams of the MLI patch. If this task were performed perfectly, the cutter would follow a linear path (for the first seam) and it would be impossible to estimate a plane using a set of collinear (or nearly collinear) points. In that case, the second component can only be estimated when the operator begins to cut a side of the patch that is orthogonal (or has a significant orthogonal component) to the first side.

The developed method first defined a sliding window for the incoming position and force measurements. Because the robot was teleoperated, the algorithm could not make any assumptions about the rate of position change; thus, the current measurement was sampled only if it was greater than a minimum distance from the last sample. This was to prevent the adverse effect of clustered data on the accuracy of later registration. The next step was to estimate the stiffness *k* of the MLI, based on the model Δ*z* = *k*Δ*f*_*z*_, where Δ*z* was the difference of adjacent position data in the direction of the cutter axis *z*_*c*_ and Δ*f*_*z*_ was the difference of the corresponding measured normal forces. If the correlation between these quantities exceeded a threshold, a least squares method was used to estimate the stiffness value *k*.

Once the stiffness was determined, the Z coordinate of every position *z*_*i*_ in the window was shifted to a common reference force, *f*_*nom*_, yielding a new set of Z coordinates ${\hat{z}}_{i}$ :

$${\hat{z}}_{i}=z_{i}+\frac{f_{nom}-f_{z}}{k}$$ (6)

The implementation set *f*_*nom*_ to the desired normal force, which was constant (i.e., not determined by the force applied by the operator via the master manipulator).

**FIGURE 8:**
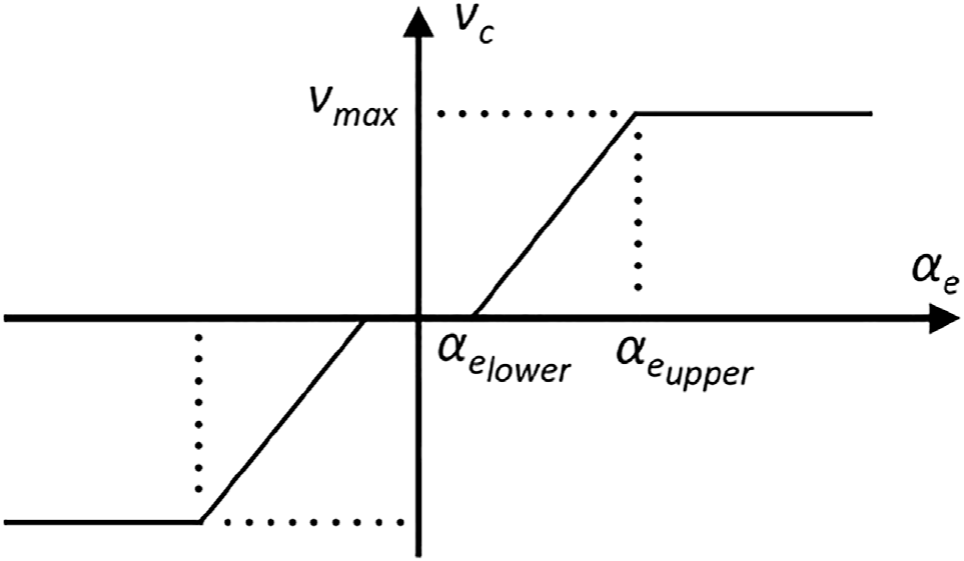
Profile of velocity correction, *v*_*c*_ as a function of estimated misalignment angle *α*_*e*_.

Finally, a principal component analysis (PCA) of the 3D cutter positions in the sliding window, $\left(x_{i},y_{i},{\hat{z}}_{i}\right)$ , was performed to determine the primary cutting direction, ${\hat{x}}_{c}$ , as defined in Figure 7A. The angle between *x*_*c*_ and ${\hat{x}}_{c}$ (denoted by *α*_*e*_ in Figure 7A) was the estimated misalignment, and the correction velocity was performed by rotating the end-effector around its local axis, *y*_*c*_, with the velocity profile shown in Figure 8. In particular, this profile included a deadband, given by $\alpha_{e_{lower}}$ , and a maximum correction velocity determined by $\alpha_{e_{upper}}$ . The correction was performed until *x*_*c*_ was aligned within $\alpha_{e_{lower}}$ of ${\hat{x}}_{c}$ , which aligned the cutter to the plane in the direction of cutting. When the operator changed the direction of motion, the method could estimate the other component of the plane normal and perform the correction accordingly.

#### 2.5.2 Online Estimation of Friction and Cutting Force

Section 2.4.1 described a task monitor that estimated the force in the direction of cutting, based on a model (Eq. 5) with a coefficient of kinetic (Coulomb) friction *μ*_*k*_ and a constant cutting force *F*_*c*_, Xia et al. (2013); Kandaswamy et al. (2014). During cutting, this task monitor compared the measured force to the force predicted by the model and could stop the task if the discrepancy was greater than a specified threshold (indicating a failure). One limitation was that these two parameters were based on off-line experimental measurements and therefore did not consider variations in the material properties of the MLI (e.g., due to long-term exposure in space). This section describes an estimator, developed by Li and Kazanzides (2015), that updated the model parameters during the task, based on sensor feedback. This introduced several design challenges. One challenge was the tradeoff between the responsiveness of the estimator and the ability to detect anomalies. For example, bunching of the tape would cause a sudden increase in the measured force, but this should be detected as an anomaly and should not allow the estimator to adapt the parameters based on that measurement. A second challenge was that the two model parameters were not observable unless there was sufficient variability in the applied normal force.

**FIGURE 9:**
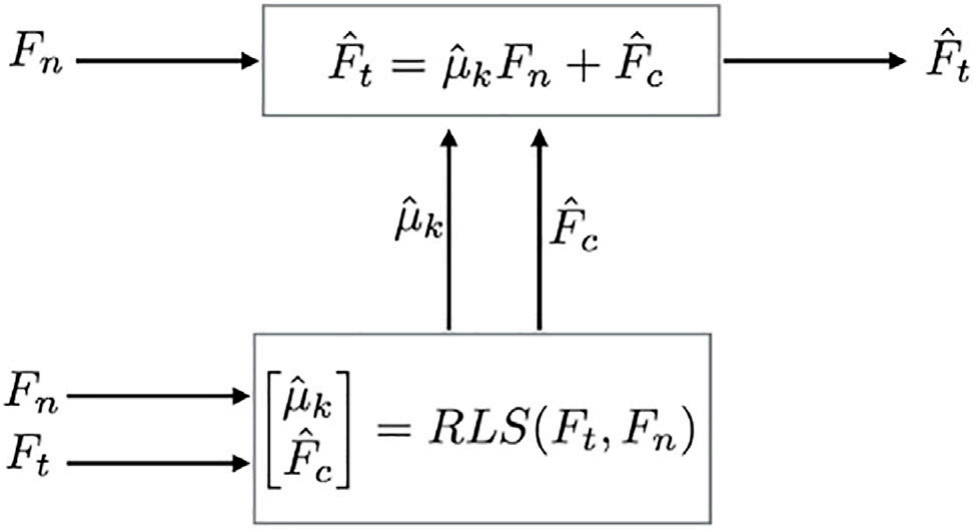
Estimator flowchart: *F*_*n*_ and *F*_*t*_ are measured by force sensor, ${\hat{\mu }}_{k}$ and ${\hat{F}}_{c}$ are estimated, and ${\hat{F}}_{t}$ is predicted by model.

The goal was to design an estimator such that for given measurements (*F*_*t*_, *F*_*n*_), parameters (*μ*_*k*_, *F*_*c*_) could be recursively estimated by ( ${\hat{\mu }}_{k}$ , ${\hat{F}}_{c}$ ) and that these estimates would adapt to small changes in the cutting environment (material properties, cutter contact conditions, etc.). But, the estimated force given by ${\hat{F}}_{t}={\hat{\mu }}_{k}F_{n}+{\hat{F}}_{c}$ should be significantly different from the measured *F*_*t*_ when a cutting abnormality occurs. This adaptive parameter update step is illustrated in Figure 9.

The proposed approach utilized a recursive least squares (RLS) estimator with vector-like forgetting factors, as described in Vahidi et al. (2005), which enable individual adjustment of the variational rates of the parameters. By incorporating forgetting factors, the estimator can be controlled such that the coefficient of kinetic friction (*μ*_*k*_) is updated taking into account more historic data and the cutting force (*F*_*c*_) is updated with more emphasis on recent data. This aligns with the expectation that if the material properties of the cutter and MLI do not change abruptly, *μ*_*k*_ should vary in a small range. Mild variations in the measured tangential force are likely due to varied cutting conditions, such as slight wrinkling of the tape, and generally do not indicate cutting failures. To handle cases like these, a lower forgetting factor was selected for *F*_*c*_ so that it can take more responsibility for adapting to the changes. The detailed mathematical derivation of the estimator is described in Li and Kazanzides (2015).

In addition, the method disabled the estimator when there was insufficient variation in the input vector (*F*_*n*_ measurements), in which case it would be impossible to estimate both *μ*_*k*_ and *F*_*c*_. This is more likely to occur when force control is used to maintain contact with the surface, as proposed in Xia et al. (2013). This check was implemented by fitting a line to the measured normal force in a sliding window. If the slope of the line was less than a specified threshold, the estimator was disabled and the parameter values were not changed. During the experiments, Li and Kazanzides (2015) also observed cases where the estimator produced negative ${\hat{\mu }}_{c}$ and/or $\hat{F_{c}}$ ; since these are physically unreasonable values, they were discarded and the previous valid estimates were used to compute the $\hat{F_{t}}$ that was used for failure detection.

## 3 Results

This section reports the results of several multi-user experiments to evaluate the model-based architecture, on ground-based test platforms, for specific satellite servicing tasks. All user studies were approved by the Johns Hopkins University Homewood Institutional Review Board (protocol HIRB00000701). The studies are reported in chronological order, beginning with model-mediated teleoperation experiments from Vozar et al. (2015a,b), followed by augmented virtuality experiments from Vagvolgyi et al. (2017, 2018); Pryor et al. (2019), and then IPSE experiments from Pryor et al. (2020). We do not report test results for individual components, such as the task monitors or model update methods, which can be found in the relevant cited papers.

For all experiments reported below, we constructed mock MLI blankets from representative (but not space-qualified) industrial materials that closely resemble the physical properties of the space-qualified MLI materials commonly employed in satellites, as described in Vozar et al. (2015a).

### 3.1 Model-Mediated Teleoperation Experiments

**FIGURE 10:**
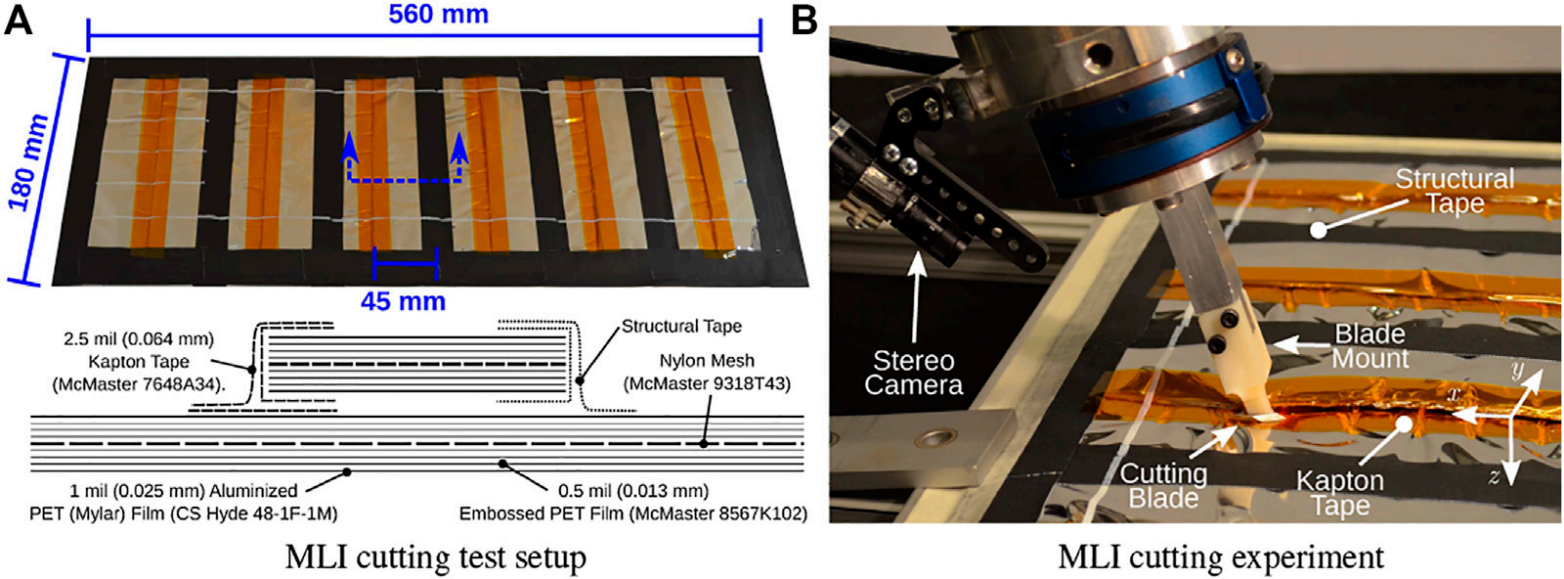
Model-mediated teleoperation experiment from Vozar et al. (2015a). **(A)** MLI taping pattern, showing six test strips. One was used for practice **(far left)**, four were used for the trials **(middle four)**, and one **(far right)** was not used unless a spare was necessary. A schematic section view shows the structure of the blanket layers. **(B)** Closeup view of the blade apparatus cutting through Kapton tape on the satellite mock-up panel.

This section summarizes results of experiments performed by Vozar et al. (2015a,b). Vozar et al. (2015a) first evaluated the plane task model, which defines a virtual fixture on the master manipulator and a hybrid position/force controller on the remote robot, as described in Section 2.3.1. For this study, 20 volunteers (9 male, 11 female), ranging in age from 18 to 27 years, were recruited from a population of graduate and undergraduate students at Johns Hopkins University. Each subject used the crescent-shaped side of the cutting blade to cut one strip of Kapton tape affixing two layers of MLI blanket, keeping the cut as straight as possible, as shown in Figure 10. Four configurations were tested, varying delay and controller type: 1) no delay, model-mediated, 2) 4 s delay, model-mediated, 3) no delay, conventional and 4) 4 s delay, conventional. For the conventional teleoperation scenario, force control on the remote robot was disabled.

Subjects were given a chance to practice with the robot system before performing the tests. For each scenario, the cutting blade was placed inside a pre-cut incision on the Kapton tape, with the blade oriented to the cutting plane. Then, the subject teleoperated the WAM robot to cut a single 140 mm line, with start-points and end-points indicated with white paint on the blanket.

Measurements included the number and type of adverse events, such as tape bunching and cutter slipping out of the seam, average velocity, path straightness, roughness of cut edges, and subjective task load based on a NASA TLX survey, Hart (2006).

The results indicated that the total failure rate was not affected by either delay or controller type, although the types of failures varied between these scenarios. The introduction of the 4 s delay reduced the mean average speed from 2.04 mm/s to 1.76 mm/s, which was statistically significant (*p* = 0.087), and decreased the straightness of the cut (mean path error from straight line) from 0.921 to 1.44 mm, which was also statistically significant (*p* < 0.001). But, there was no significant effect of controller type on either metric. The overall workload, defined as the sum of the responses to all the questions in the TLX survey, ranged from 6 (least) to 42 (most). The mean workloads with and without delay were 23.0 and 18.9, respectively, and the effect was significant (*p* < 0.001). The mean workloads with and without model-mediated teleoperation were 19.8 and 22.1, respectively, which were also significant (*p* = 0.025). Thus, the summary of the experimental results is that time delay causes operators to move more slowly and makes it more difficult to cut in a straight line, regardless of whether or not model-mediated teleoperation is employed. However, model-mediated teleoperation significantly decreases the operator workload.

Vozar et al. (2015b) subsequently reported the results of a four-subject pilot study that compared the baseline plane virtual fixture described above to configurations that added the following to the plane virtual fixture: 1) scaled axes, where motion orthogonal to the cutting direction is scaled by 25% with respect to motion along the cutting direction, 2) virtual non holonomic constraint (VNHC), where the operator steers the cutter, and 3) non holonomic virtual fixture (NHVF), which adds a soft virtual fixture line to the previous case. These latter two conditions are described in Section 2.3.1. The results indicated that the additional constraints and virtual fixtures did not appear to affect the cut speed or quality, but led to a small reduction in the reported subjective workload. However, the sample size was too small to show statistical significance.

### 3.2 Augmented Virtuality Experiments

This section briefly describes initial experiments, more fully described in Vagvolgyi et al. (2017, 2018), followed by a user study performed with trained robot operators, Pryor et al. (2019).

We first measured the augmented virtuality visualization accuracy by comparing real photos to computer generated renderings, with qualitative results shown in side-by-side images and quantitative results presented as distances between manually selected visual landmarks, Vagvolgyi et al. (2017). In particular, the mean error over 94 landmark observations was 3.95 pixels, with a standard deviation of 3.22 pixels (all cameras were approximately two megapixels). In a preliminary study, six operators used both conventional and augmented virtuality visualization while using the da Vinci console to teleoperate a robot, with no added time delay, to draw a pattern on the MLI surface of the mock satellite. The results indicated a small improvement in operator performance, leading to speculation that further system improvements may result in more significant performance gains.

After improving the system, we performed another pilot study, where seven operators used the da Vinci console to teleoperate a robot, with 5 s of telemetry time delay, to draw on the MLI hat using a rotary crayon, Vagvolgyi et al. (2018). A foam support was placed inside the MLI hat to provide sufficient rigidity for the drawing task and also to keep the MLI hat from sagging due to the effects of gravity. Once again, the conventional visualization (camera image) was compared to the augmented virtuality visualization; however, because the latter configuration included an environment model, we also provided virtual fixtures that the operator could invoke to help stay on the defined cutting path (green lines in Figure 4C). The results showed that the augmented virtuality visualization and virtual fixtures allowed operators to perform the task more quickly and accurately, with straighter paths and minimal gaps.

**FIGURE 11:**
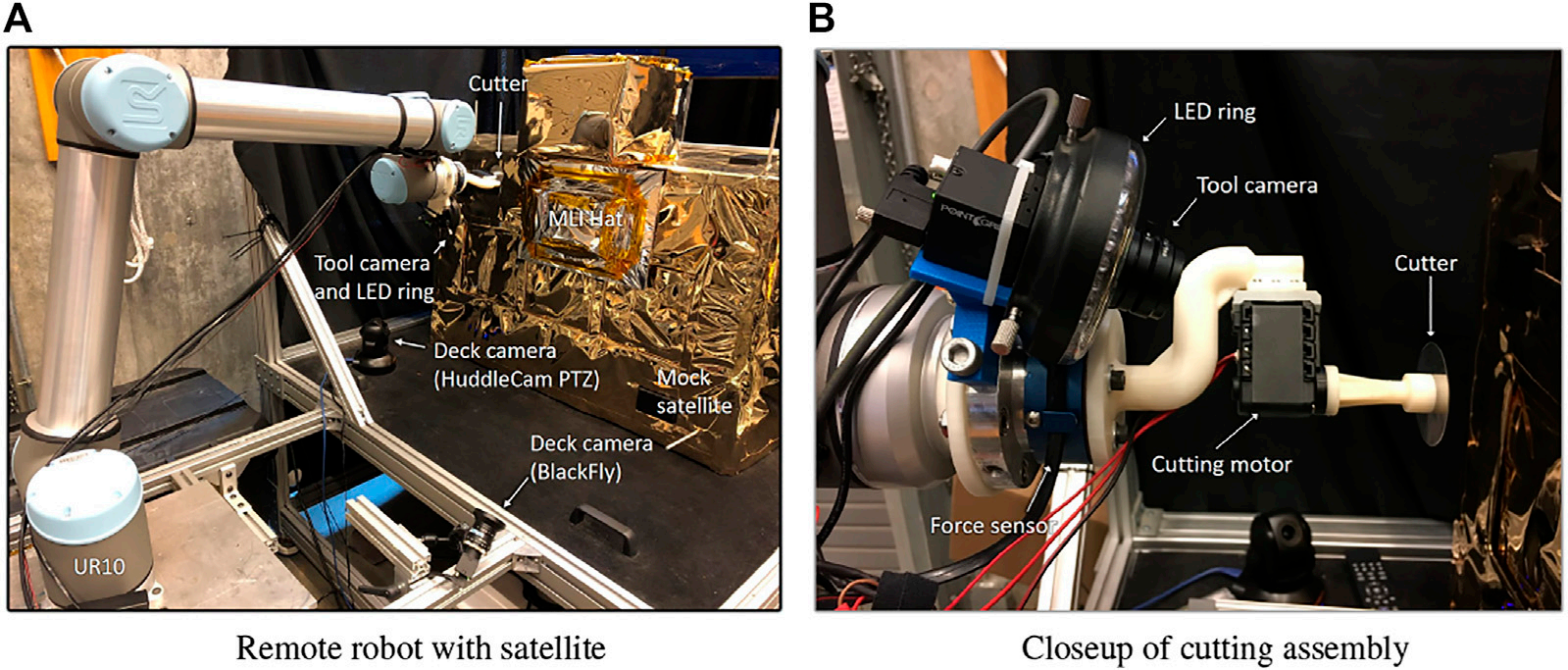
Soft-MLI setup for cutting MLI hat. **(A)** remote robot with satellite; **(B)** Closeup of cutting assembly on UR10 robot.

Finally, we performed a user study with five trained robot operators (100% of the target population), to cut two sides of an MLI hat in the Soft-MLI testbed (Figure 11), with a telemetry time delay of 5 s, Pryor et al. (2019). This testbed employs a UR-10 robot (Universal Robots, Odense, Denmark), equipped with a rotary cutting tool. The tool is composed of a 45 mm circular blade (Arteza, Wilmington DE), attached to a Dynamixel MX-12W servo motor (Robotis, Lake Forest, CA), that is mounted on a six axis force/torque sensor (JR3 Inc., Woodland, CA). A BlackFly (FLIR Integrated Imaging Solutions Inc. BC, Canada) 1080p color camera is also mounted on the UR-10 end-effector to provide a close-up view of the blade and worksite. The lens of the camera is equipped with a LED ring light. The testbed also includes one pan-tilt-zoom (PTZ) camera (HuddleCam Downingtown, PA) and one BlackFly deck camera equipped with a wide angle lens (Rochester, NY) as proxies for cameras to be mounted on the servicer spacecraft deck.

For this study, we developed a more representative conventional teleoperation interface that uses a keyboard and GUI, instead of the da Vinci master console. Thus, we had two different visualization interfaces: conventional camera view (CAM) and augmented virtuality (AV), and two different teleoperation interfaces: keyboard/GUI (KB) and da Vinci (dV). The two teleoperation interfaces are visible in Figure 1. Details about the features of each interface are described in Pryor et al. (2019).

The setup for each experimental trial consisted of a robotic image survey to build the environment model, as described in Section 2.1.1. Then, the task model (desired cut path) was defined in the same relative location on each reconstructed hat model. Each trial began with the robot in the same position relative to the mock satellite.

During trials, operators sat out of visual range of the robot, relying only on the time-delayed camera feedback for visualization. In addition, all operators wore noise-canceling headphones to prevent them from hearing real-time (i.e., undelayed) audio feedback, such as changes in the cutting motor sound. The order of trials was fixed to introduce no more than one new feature at a time. Each operator first performed the conventional (KB + CAM) trial, which emulated their familiar teleoperation interface, though with different hardware and software. Next, the augmented virtuality (AV) visualization was introduced, while keeping the familiar keyboard teleoperation interface (KB + AV). Note, however, that the keyboard interface was enhanced to take advantage of the constructed models. For example, operators could use the keyboard to command robot motion in a task frame aligned with the cutting path, so that a single degree of freedom controlled progress along that path. Finally, the AV visualization was kept and the da Vinci teleoperation interface was introduced (dV + AV). In this interface, operators could enable virtual fixtures to provide haptic guidance and/or set anisotropic gains, both of which were defined with respect to the cutting path task frame. Operators were allowed to practice with each configuration prior to beginning each trial.

**FIGURE 12:**
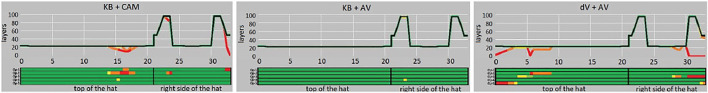
Visualization of the number of layers successfully cut in all MLI cutting trials. Horizontal axis represents cutting progress (cm), starting at the top of the hat then continuing on the right side. The thin black lines indicate the number of layers that need to be cut, and the thick colored lines show the number of successfully cut layers for each trial. A single sheet of MLI consists of 23 layers, but there are as many as 95 layers at the corners where the MLI is folded and taped multiple times. The colored horizontal bands (one for each operator) under the charts show the number of layers cut for each trial. Colors: dark green indicates all layers cut; light green indicates one layer uncut; yellow indicates two to three layers uncut; orange indicates 4–10 layers uncut; red indicates more than 10 layers uncut.

As a measure of the success of each cutting trial, Figure 12 shows the number of layers cut compared to the number of layers present, with a quantitative summary in Table 1. Note that the geometry of the hat construction causes a significant increase in the number of layers that must be cut at a corner. We assumed that the cut is likely to be successful if all layers are cut, or if only the innermost MLI layer is not cut in a short segment. The exact degree of success depends on the location of the cutting failure, the condition of MLI materials, and other factors; thus, they are determined on a case-by-case basis. The results indicate that the KB + AV configuration led to the highest percentage of complete and acceptable cuts. In addition, despite the increased number of layers, the corners typically saw more success than the straight sides. We attribute this to the additional structural integrity of the hat at the corners, which restricts the layers from spreading apart.

**TABLE 1:** Results of MLI hat cutting experiments with five trained robot operators, using conventional input and visualization (KB + CAM), conventional input and augmented virtuality visualization (KB + AV), and da Vinci master console for input and augmented virtuality visualization (dV + AV). Success rate is quantified by percentage of cut path with given number of uncut layers. Goal was to cut all layers, so the ideal result would be 100% for 0 “not cut” layers.

Not cut	KB + CAM	KB + AV	dV + AV
0	95.29%	99.71%	91.18%
1	0.00%	0.00%	0.59%
2–3	0.59%	0.29%	1.76%
4–10	2.35%	0.00%	3.24%
>10	1.76%	0.00%	3.24%

Table 2 presents the results of the post-experiment survey, where operators rated the difficulty of each system configuration on a scale from 1 (very easy) to 5 (very hard). All five operators selected the KB + AV configuration as the easiest or as one of the easiest and four operators rated the dV + AV configuration as the hardest. This is consistent with the NASA TLX results reported in Pryor et al. (2019). In addition, Table 2 shows the total time to cut the two sides of the hat. For four out of the five subjects, the conventional keyboard/GUI interface with the augmented virtuality visualization (KB + AV) took less time than with the conventional visualization (KB + CAM). However, all five subjects completed the task in the shortest time using the da Vinci interface (dV + AV). This is likely because the da Vinci’s direct teleoperation does not allow the chance to preview the result of a command, eliminating the time spent confirming that the correct command will be issued. For this reason, the lower time does not indicate an improvement.

**TABLE 2:** MLI hat cutting experiment: Post-experiment survey results (1 = very easy, 5 = very hard) and total times (seconds) required by each robot operator for each interface.

	Survey rating			Total time (sec)		
Operator	KB + CAM	KB + AV	dV + AV	KB + CAM	KB + AV	dV + AV
1	4	3	3	2,358.5	3,139.4	1,067.2
2	3	2	2	1,088.1	983.0	776.5
3	3	2	5	2,088.5	1,639.0	1,008.7
4	3	2	4	3,154.8	1,912.8	1,162.5
5	3	3	4	4,620.2	2,846.6	1,298.0
Mean	3.2	2.4	4.0	2,662.0	2,104.2	1,062.6

In summary, the trained robot operators preferred the augmented virtuality visualization over the conventional visualization, but they preferred a conventional keyboard/GUI control interface over the da Vinci master console. This choice also aligns with their primary goal, which is to successfully complete the task.

### 3.3 Interactive Planning and Supervised Execution (IPSE) Experiments

**FIGURE 13:**
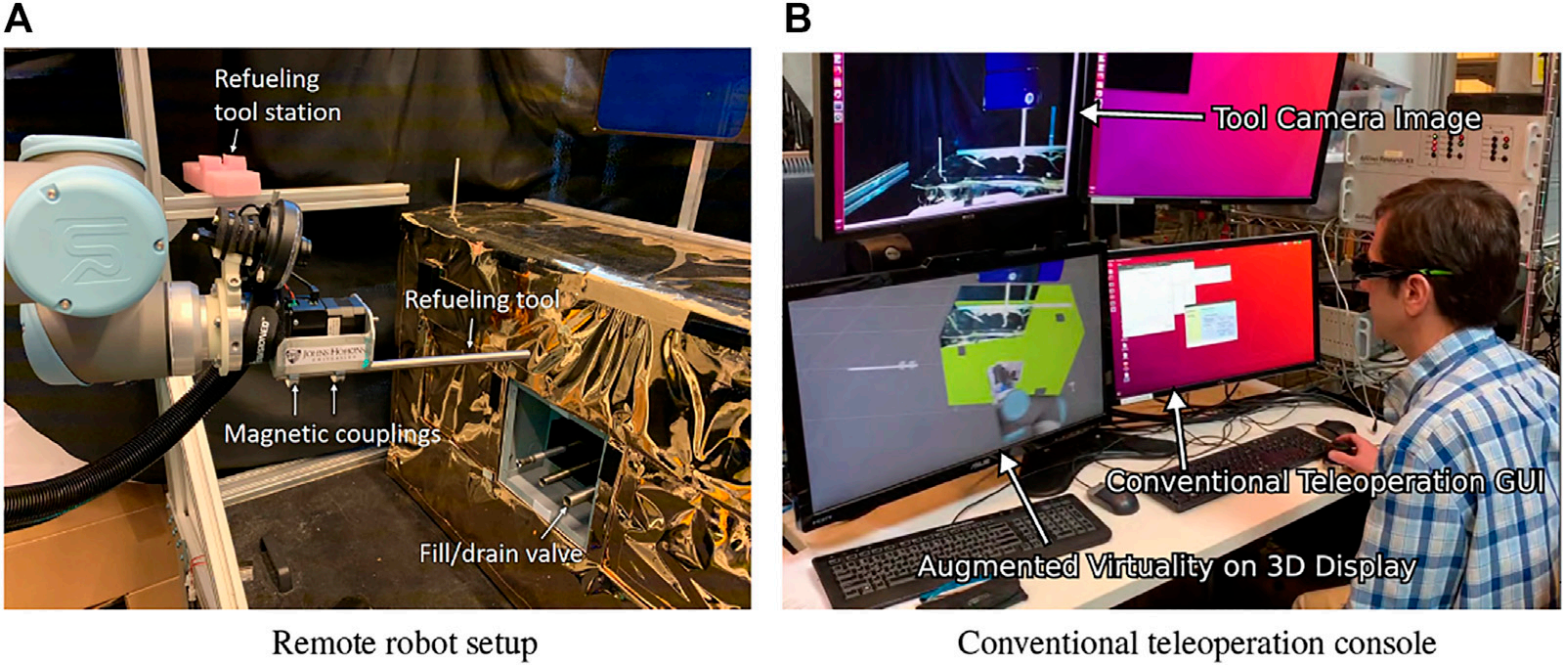
IPSE test setup. **(A)** Space-side setup with mock satellite, mock servicing robot, and refueling tool station; **(B)** Conventional teleoperation console with 3D monitor for augmented virtuality visualization (note that operator is wearing shutter glasses).

The IPSE module was evaluated in the Soft-MLI setup, with the mock servicing robot and satellite shown in Figure 13A, Pryor et al. (2020). Each experiment began with the environment model already created, as described in Section 2.1.1. The operator first performed the *tool pickup* task by commanding the robot to the refueling tool station and lowering it onto the tool (a section of tube with outer diameter 0.500″) to engage the magnetic attachment. Once at least one magnet attached, an experimenter recorded whether the tool was aligned correctly and manually aligned the tool if necessary. The operator then performed the *refueling* task by commanding the robot to move from the refueling tool station to a tube, with inner diameter 0.584”, on the mock satellite, which represents the fuel port, and then inserting the tool at least 3 cm into the tube. Each operator performed the experimental task three times, in each of the following planning configurations: 1) Conventional teleoperation console (Figure 13B), with ability to preview robot position goal entered into GUI as a yellow ring on the 3D monitor (better seen in Figure 4C), 2) Conventional teleoperation console, with IPSE 2D interface (on RViz), and 3) da Vinci teleoperation console, with IPSE 3D interface. In all configurations, the execution phase was performed on the conventional teleoperation console, with the augmented virtuality visualization on a 3D monitor with shutter glasses. For the first configuration, it was also possible to use a 3D mouse to move the robot.

Although IPSE was designed to enable the operator to switch between the 2D and 3D interfaces at will, we decided to evaluate them separately for two reasons: 1) to compare their effectiveness in performing the task, and 2) to ensure that each interface was actually used. Note that because the 3D interface did not fully support all functionality, operators were allowed to use the 2D interface in cases where the 3D interface did not provide the necessary functionality (e.g., to modify the motion speed). In addition, the 2D interface was used to initiate execution because the augmented virtuality visualization had not yet been implemented on the da Vinci console.

Six operators were recruited from a population familiar with teleoperation, including use of the da Vinci surgical system, to reflect the fact that this task would be performed by trained operators. We categorized the results for the two tasks, *tool pickup* and *refueling*, into three categories: full success, partial success, and failure. Partial success was defined as an attached but improperly aligned tool in the tool pickup task, and as a tool inserted less than the desired 3 cm in the refueling task. For both tasks, failure was indicated when the operator believed it was no longer possible to complete the task.

Table 3 summarizes the key results from the experiments. The *tool pickup* task had no failures under any of the three conditions, which we believe reflects the fact that the magnetic mount is sufficiently strong to attach even across a fairly large distance. However, attaching at a distance increases the probability of a misaligned tool, which was evident in the partial success rate. Using conventional teleoperation, two of the operators misaligned the tool. Success rates for the *refueling* task were much lower, demonstrating the significantly higher requirement for precision in this task due to the clearance of 2.13 mm (0.084”) between the tool and tube. Of the five failures across all conditions, four were due to the operator knocking the tool off the mount by contacting an obstacle in the environment. Of these, three were caused by contacting the refueling tool holder, which was visible when the operators were introduced to the task but was not modeled in the virtual environment. In addition, this task affords the opportunity to dislodge the magnetically attached tool without knocking it off entirely, and the number of such misalignments (of the fully or partially successful tasks) is also reported in Table 3. Two of these misalignments were also due to contacting the refueling tool holder. It appears that the promise of the virtual environment and/or collision detection may have been detrimental to overall performance because operators expected that every collision would be visible in the virtual environment or detected by the IPSE system.

**TABLE 3:** IPSE experimental results for *tool pickup* and *refueling* tasks, indicating number of trials (of 6 for each test configuration) with full success (S), partial success (P), and failure (F); number of successful trials with tool misalignment (Mis.) in refueling task; task times (*min:sec*) and user ratings (1-5, in order of increasing difficulty), in format *mean (standard deviation)*.

Test	Tool pickup				Refueling					User
Config	S	P	F	Time	S	P	F	Mis	Time	Rating
Conv	4	2	0	11:31 (4:17)	3	1	2	2	17:35 (7:44)	3.5 (0.6)
IPSE 2D	6	0	0	6:34 (4:01)	5	1	0	1	12:18 (5:27)	1.7 (0.8)
IPSE 3D	6	0	0	10:59 (4:38)	3	0	3	1	30:22 (4:01)	4.5 (0.6)

The combined success rate for the IPSE-3D interface (50%) was the lowest of the three conditions, followed by the conventional interface (67%). Only the IPSE-2D interface had no failures, and it also had the highest full success rate of the three. While the IPSE-2D interface improved task performance, the IPSE-3D interface led to worse results than the conventional interface. We also asked the participants to rate the difficulty on a scale of 1–5, where higher numbers indicate greater difficulty. The results correlate with the performance measures: The conventional interface had a mean rating of 3.5 (standard deviation 0.6), the IPSE 2D interface was rated 1.7 (0.8), and the IPSE 3D interface was rated 4.5 (0.6).

Although execution time is significantly less important than success rate, Table 3 also reports the time to successful completion of each task. The results for the conventional and IPSE-2D cases show that the IPSE-2D interface allowed operators to complete the task faster, which we attribute to the lower difficulty and the operators’ increased confidence in their ability to safely execute longer motions. The results for the IPSE-3D interface, however, indicate that in the less-constrained tool pickup it was comparable to the conventional interface, but in the severely constrained refueling task it required much longer execution times than the other interfaces.

## 4 Discussion

In a conventional teleoperation system, the operator views images from one or more remote cameras and uses input devices to send motion commands to the remote system. When the remote system is in space, telemetry delays can increase the level of difficulty and cause operators to adopt strategies such as “move and wait”. While increased telemetry delay is perhaps the most obvious challenge, limited situation awareness, due to sparse or suboptimal camera views, may be an even bigger concern, especially given payload constraints that limit the number and placement of cameras. We described a model-based architecture to enable semi-autonomous teleoperation with improved visualization, control and monitoring and we summarized key components that we developed over the last 10 years.

We performed several user studies to evaluate teleoperation systems composed from different components of the overall model-based architecture. We found that the most significant improvement was obtained by enhancing the operators’ situation awareness, via the augmented virtuality visualization described in Section 2.2.2, as well as by improving their ability to precisely specify intended motion, which were both evaluated by trained robot operators in Section 3.2. In contrast, we found it more challenging to significantly improve the control interface, whether through model-mediated teleoperation or through an immersive 3D console such as the da Vinci master console. The model-mediated teleoperation, using virtual planes and lines in the Firm-MLI setup, generally reduced the task load when performing the experiments reported in Section 3.1, but did not significantly improve task performance. In some cases, operators appeared to be working against the virtual fixture, Vozar et al. (2015b), so it is possible that a different implementation (for example, different gains for the NHVF controller) would have produced better results. However, the study with the trained robot operators provided evidence that small changes to the familiar control interface, such as allowing the keyboard to command motion with respect to the task frame, could improve task performance. One limitation of this study is that it did not evaluate the relative benefits of the augmented virtuality visualization and the ability to command in the task frame, though the important point is that both rely on the creation of the environment and task models described in Section 2.1.

While the trained robot operators preferred the augmented virtuality visualization over conventional visualization, they were willing to sacrifice this feature to keep their conventional keyboard/GUI control interface, rather than have to use the da Vinci console. These operators have trained for years using the conventional interface and thus it is not surprising that they would find it more challenging to use the significantly different da Vinci interface. On the other hand, our experiments with the interactive planning environment, described in Section 3.3, also found that the da Vinci interface was more difficult to use, even though the subjects in those experiments were more familiar with that interface. We believe this outcome was due to several factors. First, some tasks, such as cutting MLI in a straight line, do not benefit from the ability to command motion in 6 DOF; in fact, this flexibility could be detrimental to task performance and we therefore incorporated virtual fixtures to restrict motion along some degrees of freedom. Another limitation was that the da Vinci interface did not support specification of precise motions with respect to identified features (task frames) in the environment model. For example, in the interactive planning experiments presented in Section 3.3, operators were able to use the keyboard to move the refueling tool with respect to a task frame affixed to the tube emulating the fuel port. Similarly, in the MLI cutting experiments, the operators could use the keyboard to move the cutter with respect to the desired cutting path. Anecdotally, in the interactive planning study, several operators found the 3D interface well suited for planning larger motions, such as first moving to the refueling tool station and then moving from there to the vicinity of the fuel port. Our conclusion, therefore, is that it is best to offer multiple interfaces so that operators can choose the best interface for a particular task step.

The model-based framework also enables the system to more effectively incorporate sensor feedback that is not used for robot control, either to update the models or to monitor task performance (including error detection). We presented several examples, including task monitors based on measuring force (Section 2.4.1) or estimating force from vision (Section 2.4.2), both in position-controlled directions, and model updates based on measured position in the direction of force control (Section 2.5.1). But, it is important to note that different system configurations are possible. For example, the estimated cutting blade engagement force (Section 2.4.2) could be used for feedback control, instead of as a task monitor, in which case the measured position in that direction could be used either to monitor the task or update the model. In addition, in some cases it is possible to use combinations of sensor feedback to satisfy multiple goals, such as when using the measured normal force to control sliding along a surface, the measured tangential force as a task monitor, and both force components to update the cutting force model (Section 2.5.2).

Finally, it is important to acknowledge that this work assumed communication latencies on the order of seconds and that it was feasible, and therefore preferable, for human operators to perform the considered satellite servicing tasks. In addition, mission considerations placed a premium on avoiding failure, rather than on other factors such as optimizing time. This may also explain the preference for a keyboard interface, where operators can specify precise intended motions, rather than a 3D joystick or mouse, where it is possible to obtain imprecise or unintended motions. These considerations may not apply in other scenarios. For example, 3D (joystick) control may be preferable for tasks that require higher dexterity and/or faster completion times, and have some tolerance for imprecise motion. Alternatively, scenarios with larger time delays or tasks that require response times that are faster than the communication delay, may require a higher level of autonomy. Nevertheless, within the continuum between direct teleoperation and full autonomy, we believe that our model-based architecture can provide benefits in visualization, control and error detection for robotic manufacturing, assembly, and servicing of in-space assets.

## Data Availability

The data analyzed in this study is subject to the following licenses/restrictions: The datasets presented in this article are not readily available because they were collected under an institutional review board protocol. Requests to access these datasets should be directed to Peter Kazanzides, pkaz@jhu.edu.

## Abbreviations

AR, Augmented Reality; AV, Augmented Virtuality; CAD, Computer-Aided Design; CAM, Camera-based visualization; DOF, Degrees Of Freedom (of a robot); dV, da Vinci Surgical System; GPU, Graphics Processing Unit; GUI, Graphical User Interface; HSV, Hue-Saturation-Value color model; IPSE, Interactive Planning and Supervised Execution; KB, Keyboard (and mouse) input; LED, Light Emitting Diode; MLI, Multi-Layer Insulation (encases a satellite); MTM, Master Tool Manipulator of da Vinci Surgical System; NHVF, Non Holonomic Virtual Fixture; PD, Proportional-Derivative controller; TLX, NASA Task Load Index; VNHC, Virtual Non Holonomic Constraint; VR, Virtual Reality.
